# Genotype-phenotype correlation and management of Megacystis-Microcolon-Intestinal Hypoperistalsis Syndrome: a descriptive cohort study

**DOI:** 10.1186/s13023-025-04154-9

**Published:** 2025-12-12

**Authors:** Johannes Hilberath, Ilias Tsiflikas, Anna Sanders, Justus Lieber, Tobias Luithle, Tobias B. Haack, Ekkehard Sturm, Jörg Fuchs, Steven Warmann, Christoph Slavetinsky

**Affiliations:** 1 Pediatric Gastroenterology and Hepatology, University Children´s Hospital Tübingen, Tübingen, Germany; 2 Center for Rare Diseases, University of Tübingen, Tübingen, Germany; 3 Department of Diagnostic and Interventional Radiology, University Hospital of Tübingen, Tübingen, Germany; 4 Department of Pediatric Surgery and Urology, University Children´s Hospital Tübingen, Hoppe-Seyler-Straße 3, D-72076 Tübingen, Germany; 5 Institute of Medical Genetics and Applied Genomics, University of Tübingen, Tübingen, Germany; 6 Pediatric Surgery, Charité University Hospital, Berlin, Germany; 7 Cluster of Excellence EXC 2124 Controlling Microbes to Fight Infections, Tübingen, Germany

**Keywords:** Megacystis-Microcolon-Intestinal Hypoperistalsis Syndrome, Intestinal failure, Pediatric intestinal pseudo-obstruction (PIPO), *ACTG2*, *MYH11*, *MYLK*, *LMOD1*

## Abstract

**Background:**

Megacystis-Microcolon-Intestinal Hypoperistalsis Syndrome (MMIHS) is a rare genetic visceral myopathy, with a historically high mortality rate. Its genetic and phenotypic variability and management options remain poorly characterized. This study correlates genotype with phenotype and subsequently analyzes treatment and outcome of patients with pediatric-onset MMIHS.

**Results:**

We retrospectively analyzed 26 MMIHS patients (median age 97 months, 62% female) with molecular diagnostics in 19 patients at a German quaternary intestinal rehabilitation center followed between 2012 and 2025. *ACTG2* (15/19) was the most common causative gene variant, followed by *MYH11* (2/19), *MYLK* (1/19), and *LMOD1* (1/19). Megacystis was present in 96% (all detected prenatally), intestinal hypoperistalsis in 100%, and microcolon in 57%. High rates of proximal intestinal stenosis (35%), mal-/non-rotation (39%), IFALD (58%), and Cholelithiasis (65%) were observed. All patients experienced clinical (sub)ileus, with 85% requiring ostomy and parenteral nutrition, key determinants of unfavorable outcome. Notably, *ACTG2* variants at P39 or R40 were significantly associated with more favorable outcome, evading these measures, in contrast to variants at R63, R178 or R257 (*p* ≤ 0.01). Overall long-term survival in our cohort was 88%.

**Conclusions:**

In this second-largest pediatric MMIHS cohort worldwide, genotype correlated with severity and outcomes, with *ACTG2* P39/R40 variants linked to better prognosis. Frequent occurrences of proximal intestinal stenosis, mal/non-rotations, and cholelithiasis were identified, findings that have thus far been underestimated in clinical assessments. Prenatal megacystis enables presumptive diagnosis for MMIHS, which should prompt early molecular diagnostics and genotype-guided management. Individualized care at a multidisciplinary intestinal rehabilitation center resulted in 88% long-term survival.

## Background

Megacystis-Microcolon-Intestinal Hypoperistalsis syndrome (MMIHS) is an extremely rare and severe genetic smooth muscle myopathy that primarily affects the bladder and intestine [1]. Since the first description of MMIHS in five female neonates in 1976 by Berdon et al., only 762 cases have been reported predominantly in case reports worldwide (1976–2023) [2, 3].

Affected patients are characterized by the hallmark symptoms of an enlarged, non-obstructed urinary bladder, microcolon, and defective intestinal motility. MMIHS is a known cause of pediatric intestinal pseudo-obstruction (PIPO), a heterogeneous group of most severe motility disorders [4, 5]. Patients with MMIHS often require multiple intestinal surgeries, such as placement of intestinal decompression stomas, long-term parenteral nutrition (PN), and individualized bladder management (e.g. catheterization, cystostomy) [4, 6]. The mortality rate for patients with MMIHS has improved in recent decades, but outcome is still poor with a 10-year survival rate of approximately 57% for patients diagnosed with MMIHS [6, 7]. Intestinal or multivisceral transplantation is considered the only definitive treatment for patients with MMIHS [8]. A recent case series reported 11 transplanted patients among 22 patients with MMIHS who were alive at the time of the study [1]. However, intestinal transplantation should be reserved for patients with life-threatening intestinal failure-associated complications, as rates of morbidity and mortality are high [9, 10].

MMIHS is a genetic disease induced by mutations in smooth muscle-related genes, with evidence supporting both autosomal recessive and autosomal dominant inheritance [1, 3]. Most patients harbor mutations in *ACTG2*, which encodes the enteric smooth muscle actin γ-2 and variants exhibit an autosomal dominant inheritance in many cases, though not universally [3]. Specific other genes have been implicated in causing recessive or compound heterozygous forms of MMIHS in single cases, including *LMOD1*, *MYLK*, *MYH11*, *MYL9* and *PDCL3* [11, 12]. Cases of confirmed parenteral consanguinity have been reported [1]; However, most cases arise from sporadic de-novo mutations, especially since the vast majority of patients exhibit *ACTG2* variants. Recurrent missense mutations for arginine substitutions in *ACTG2* were found to cause varying degrees of severity in the largest MMIHS case series, so far [13]. However, due to the rarity of MMIHS, the spectrum of genetic and phenotypic variability remains poorly understood. Most information regarding its clinical presentation, treatment approaches, and patient outcomes is limited to case reports or series, and exhibits drastic mortality rates [14, 15].

This study aimed to (i) analyze the phenotypic characteristics, (ii) correlate genotype with phenotype in *ACTG2* variants, and (iii) evaluate the medical, nutritional and surgical management and subsequent outcomes of patients with MMIHS treated at our intestinal rehabilitation center.

## Methods

This was a retrospective, single-center chart analysis of patients treated or evaluated for MMIHS at our intestinal rehabilitation center. All patients with confirmed genetic or working diagnoses of pediatric-onset MMIHS who were followed up between 2012 and 2025 were included in the study.

The collected data included the patients’ basic characteristics (age, sex, MMIHS-causing gene variant, and last follow-up), presence of disease-associated signs and symptoms (e.g., megacystis, microcolon, defective peristalsis), genetic screening results, surgical management, nutrition, complications, and overall outcome. If performed, molecular analysis was performed using targeted *ACTG2* next-generation sequencing in suspected cases or by exome panel diagnostics during work-up in children with PIPO. Patients with intestinal failure and a history of liver fibrosis, persistent conjugated hyperbilirubinemia, or steatohepatitis were diagnosed with IFALD if other causes of hepatopathy were excluded [16].

The parenteral nutrition dependency index (PNDI) was calculated as the ratio of non-protein energy intake (NPEI) to resting energy expenditure (REE, estimated using the Schofield Eq. 1985 [17]): PNDI [%] = (NPEI/REE) x 100. A PNDI of >120%, 80–120% and < 80% is considered very high, high, and mild, respectively, and weaning should be considered when the PNDI is below 50% [18].

To correlate genotype with phenotype, patients with *ACTG2* variants were divided into groups according to the underlying gene variants. In addition, to evaluate for long-term complications in order to provide meaningful genetic counselling, two clinical outcome categories were defined: favorable (no parenteral nutrition and no intestinal stoma) and unfavorable (parenteral nutrition and intestinal stoma). Descriptive statistical analyses were performed, and groups were compared using Fisher´s exact test and Mann-Whitney U-Test (IBM^®^ SPSS^®^ Statistics 28, IBM, Armonk, New York, USA). Analyses were performed using two-tailed tests, with p-values < 0.05 regarded as statistically significant.

## Results

### Clinical characteristics of the MMIHS cohort

**Fig. 1:**
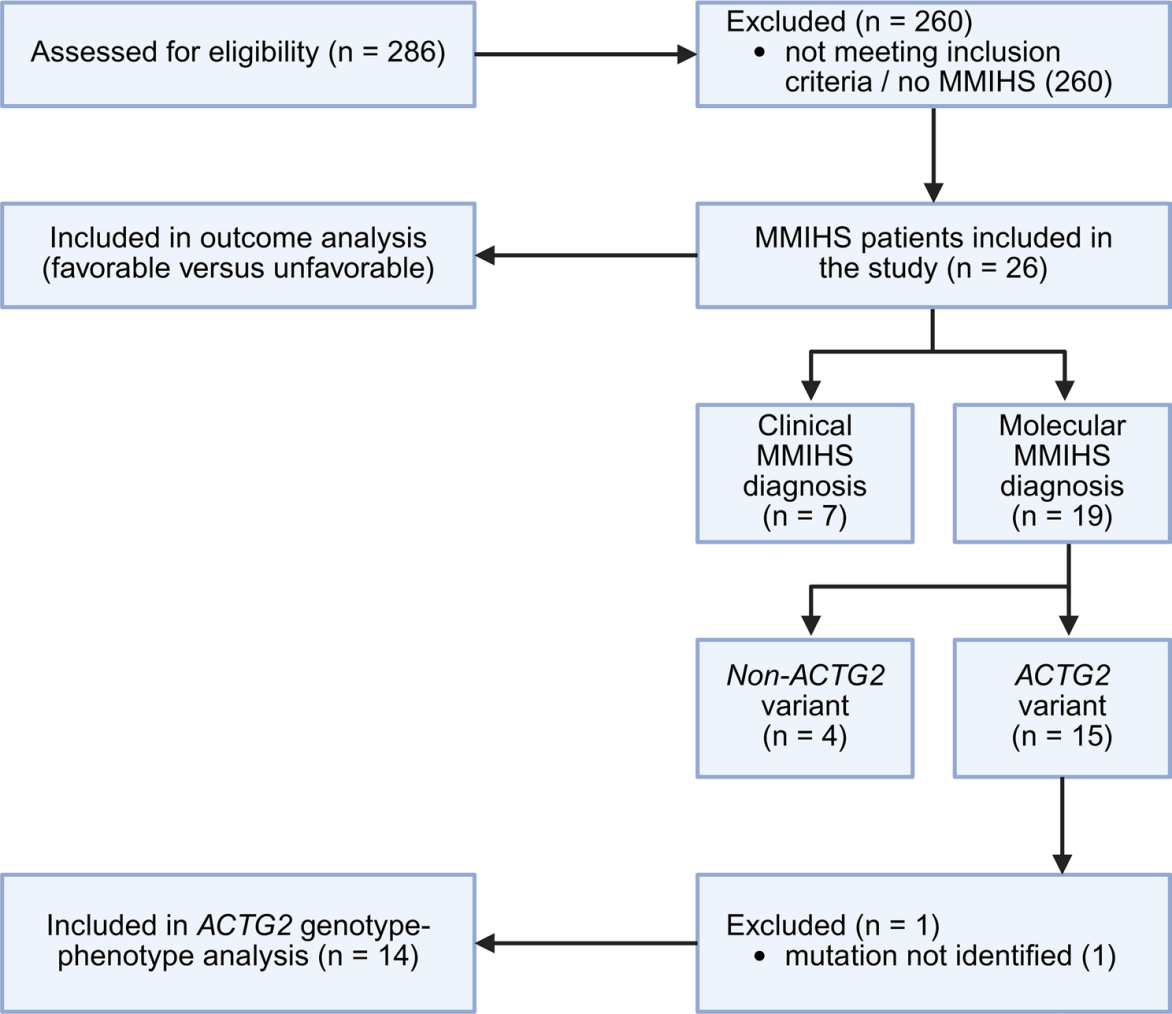
Flowchart of patient inclusion and analysis. Created with BioRender.com

Between 2010 and 2025, 286 patients with chronic intestinal failure were treated in our pediatric intestinal rehabilitation program. 26 patients were diagnosed with MMIHS in compliance with the inclusion criteria and their data were subsequently analyzed (Fig. 1). The median age at each patient’s last follow-up was 97 months (range 6-287 months), with a predominance of female patients with 62% (16/26) (Table 1).

**Table 1:** Patient characteristics of the studied MMIHS cohort

#	Age [mo]	Sex	Gene	Gene variant	Pathological prenatal US	Megacystis	Microcolon	Proximal intestinal stenosis	Disorder of intestinal rotation	Abdominal Surgery	Decompressing ostomy	Cholelithiasis	(Sub)-Ileus episodes	PN	PNDI [%]	Hepatopathy / IFALD	Death or transplantation
1	145	F	*ACTG2*	c.119G > A; p.R40H	Yes (MC, HN)	Yes	No	No	No	No	No	No	Yes	No	N/D	No	No
2	161	F	*ACTG2*	c.116 C > G; p.P39R	Yes (MC)	Yes	No	Yes (Dd)	No	No	No	No	Yes	No	N/D	No	No
3	97	F	*MYH11*	c.5630 A > G; p.E1887G	Yes (HN, MU)	No	No	No	Yes	Yes	Yes (CS)	No	Yes	Yes	50.7	Yes	No
4	176	M	*ACTG2*	c.770G > A; p.R257H	N/D	Yes	No	Yes (Jj)	No	Yes	Yes (GS, IS)	Yes (CHE)	Yes	Yes	107.1	Yes	No
5	156	F	Ø MDx	N/D	N/D	Yes	N/D	No	Yes	Yes	Yes (GS, JS)	Yes	N/D	Yes	126.8	Yes	Death
6	32	M	Ø MDx	N/D	Yes (MC)	Yes	N/D	Yes (Jj)	No	Yes	Yes (IS)	Yes	Yes	Yes	143.8	Yes	No
7	287	F	Ø MDx	N/D	N/D	Yes	Yes	Yes (Dd)	No	Yes	Yes (GS, JS)	Yes	N/D	Yes	152.7	Yes	No
8	46	M	*ACTG2*	c.532 C > T; p.R178C	Yes (MC)	Yes	N/D	Yes (Dd)	Yes	Yes	Yes (GS, IS)	Yes	Yes	Yes	108.7	Yes	No
9	99	F	*ACTG2*	c.769 C > T; p.R257C	Yes (MC, dilated bowel loops)	Yes	Yes	Yes (Dd)	No	Yes	Yes (IS)	No	Yes	Yes	109.8	Yes	No
10	97	M	Ø MDx	N/D	N/D	Yes	Yes	No	No	Yes	Yes (GS, IS)	No	Yes	Yes	129.6	No	No
11	134	M	*ACTG2*	c.770G > A; p.R257H	Yes (HN, MU)	Yes	No	No	No	Yes	Yes (GS, IS)	Yes	Yes	Yes	73.5	No	No
12	58	F	*MYLK*	c.4820G > T; p.G1607V	Yes (MC)	Yes	Yes	Yes (Dd)	No	Yes	Yes (JS)	Yes	Yes	Yes	109.3	Yes	No
13	185	M	Ø MDx	N/D	Yes (MC, dilated bowel loops)	Yes	Yes	No	No	Yes	Yes (IS)	No	Yes	Yes	56.8	Yes	No
14	44	F	*ACTG2*	N/D	Yes (MC)	Yes	Yes	No	No	Yes	Yes (IS)	No	N/D	Yes	99.8	No	No
15	176	F	Ø MDx	N/D	Yes (MC)	Yes	N/D	No	No	Yes	Yes (GS, IS)	Yes (CHE)	Yes	Yes	55.1	No	No
16	123	M	*ACTG2*	c.769 C > T; p.R257C	Yes (MC)	Yes	No	No	No	Yes	Yes (IS)	Yes	Yes	Yes	84.1	Yes	No
17	136	F	Ø MDx	N/D	Yes (MC, HN, MU, bowel obstruction)	Yes	Yes	Yes (Jj)	No	Yes	Yes (JS)	Yes (CHE)	Yes	Yes	N/D	No	ITx, Death
18	55	F	*ACTG2*	c.532 C > T; p.R178C	Yes (MC)	Yes	Yes	No	Yes	Yes	Yes (JS)	Yes (CHE)	Yes	Yes	102.6	Yes	No
19	92	M	*ACTG2*	c.770G > A; p.A257H	Yes (MC)	Yes	Yes	No	No	Yes	Yes (GS, IS)	Yes	Yes	Yes	77.6	Yes	No
20	11	F	*ACTG2*	c.532 C > T, p.R178C	Yes (MC)	Yes	Yes	No	Yes	Yes	Yes (JS)	Yes	Yes	Yes	107	Yes	Death
21	22	M	*LMOD1*	N/D	Yes (MC, dilated stomach)	Yes	No	No	Yes	Yes	No	Yes	Yes	No	N/D	No	No
22	42	F	*ACTG2*	c.769 C > T; p.R257C	N/D	Yes	No	No	No	Yes	Yes (IS)	No	Yes	Yes	100.9	No	No
23	6	F	*ACTG2*	c.188G > A; p.R63Q	Yes (MC)	Yes	Yes	No	Yes	Yes	Yes (JS)	Yes	Yes	Yes	101.8	Yes	No
24	156	M	*ACTG2*	c.769 C > T; p.R257C	N/D	Yes	N/D	No	Yes	Yes	Yes (GS)	Yes (CHE)	Yes	Yes	114.9	No	No
25	23	F	*MYH11*	c.379 C > T; pP127S	Yes (MC)	Yes	Yes	Yes (Dd)	Yes	Yes	Yes (GS, IS)	Yes	Yes	Yes	112.1	Yes	No
26	13	F	ACTG2	c.119G > A; p.R40H	Yes (MC, dilated stomach)	Yes	No	No	No	No	No	No	Yes	No	N/D	No	No

Mo, months. F, female. M, male. MC, megacystis. MDx, molecular diagnostics. HN, hydronephrosis. MU, megaureter. US, ultrasound. Dd, duodenal. Jj, jejunal. CS, cecostomy. GS, gastrostomy. IS, ileostomy. JS, jejunostomy. CHE, cholecystectomy. PN, parenteral nutrition. PNDI, parenteral nutrition dependency index. N/D, not determined/no data. IFALD, intestinal failure-associated liver disease. ITx, intestinal transplantation

**Fig. 2:**
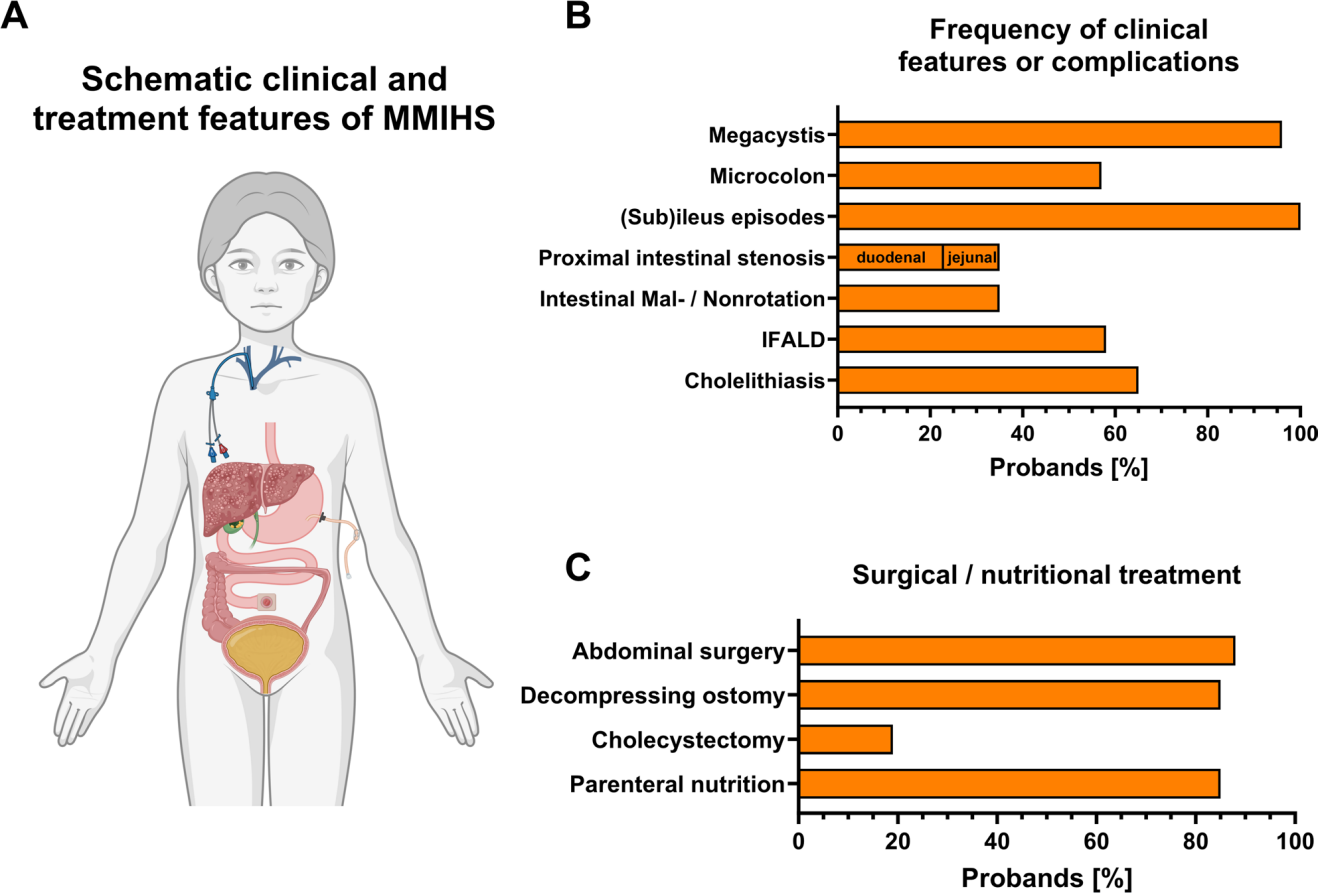
Major clinical features and treatment measures of the studied MMIHS cohort. (**A**) Disease-specific schematic features found in the cohort of MMIHS. Created using https://BioRender.com. (**B**) Frequency of clinical features or complications in the Tübingen MMIHS cohort. Respective feature is indicated together with percentages of affected patients (*n* = 26). (**C**) Surgical or nutritional treatment required and applied for the patients of the Tübingen MMIHS cohort in percentage of patients (*n* = 26)

Of the eponymous symptoms (Fig. 2A), megacystis was present in almost all patients (96%), whereas a microcolon was confirmed in only 57% (Fig. 2B). All patients in our cohort experienced recurrent clinical (sub)ileus episodes as an expression of defective intestinal peristalsis (Fig. 2B). Of note, the results of prenatal ultrasound could be retrospectively analyzed in 20 patients. All of them exhibited abnormal findings on prenatal ultrasound (Table 1), which were present in all outcome scenarios of this cohort. Megacystis was the most common prenatal finding, followed by intestinal dilatation, hydronephrosis, and megaureter (Table 1). Despite abnormal prenatal ultrasound findings, two of these children were diagnosed late with MMIHS (*ACTG2* variants) at 10 and 12 years of age. Both patients exhibited favorable outcomes. Imaging identified a high rate of intestinal malrotation or non-rotation in nine patients (39%) (Fig. 2B). Of note, 9 patients were diagnosed with proximal intestinal stenosis in either the duodenum or jejunum using endoscopy or imaging (35%), which has not been reported as a frequent finding in MMIHS, so far (Fig. 2B). The stenosis was not related to adhesions, and six of these patients required surgical intervention. A decompressing stoma (predominantly gastrostomy plus jejunostomy or ileostomy) was required and surgically placed in 85% of the patients (Fig. 2C; Table 1). Taken together, 88% of patients underwent abdominal surgery in lifetime (Fig. 2C). Apart from the four children with complete bowel continuity, all patients were dependent on home-parenteral nutrition (PN), with a median PN dependency index (PNDI) of 107% (range 50.7-152.7%) (Fig. 2C; Table 1). Cholelithiasis was found in 17 patients (65%), five of whom underwent cholecystectomy for symptomatic gallstones (Fig. 2B and C). Intestinal failure-associated liver disease (IFALD) was diagnosed in 15 patients (58%), all of whom were dependent on parenteral nutrition (Fig. 2C; Table 1).

### Genetic variants found in the MMIHS cohort

**Fig. 3:**
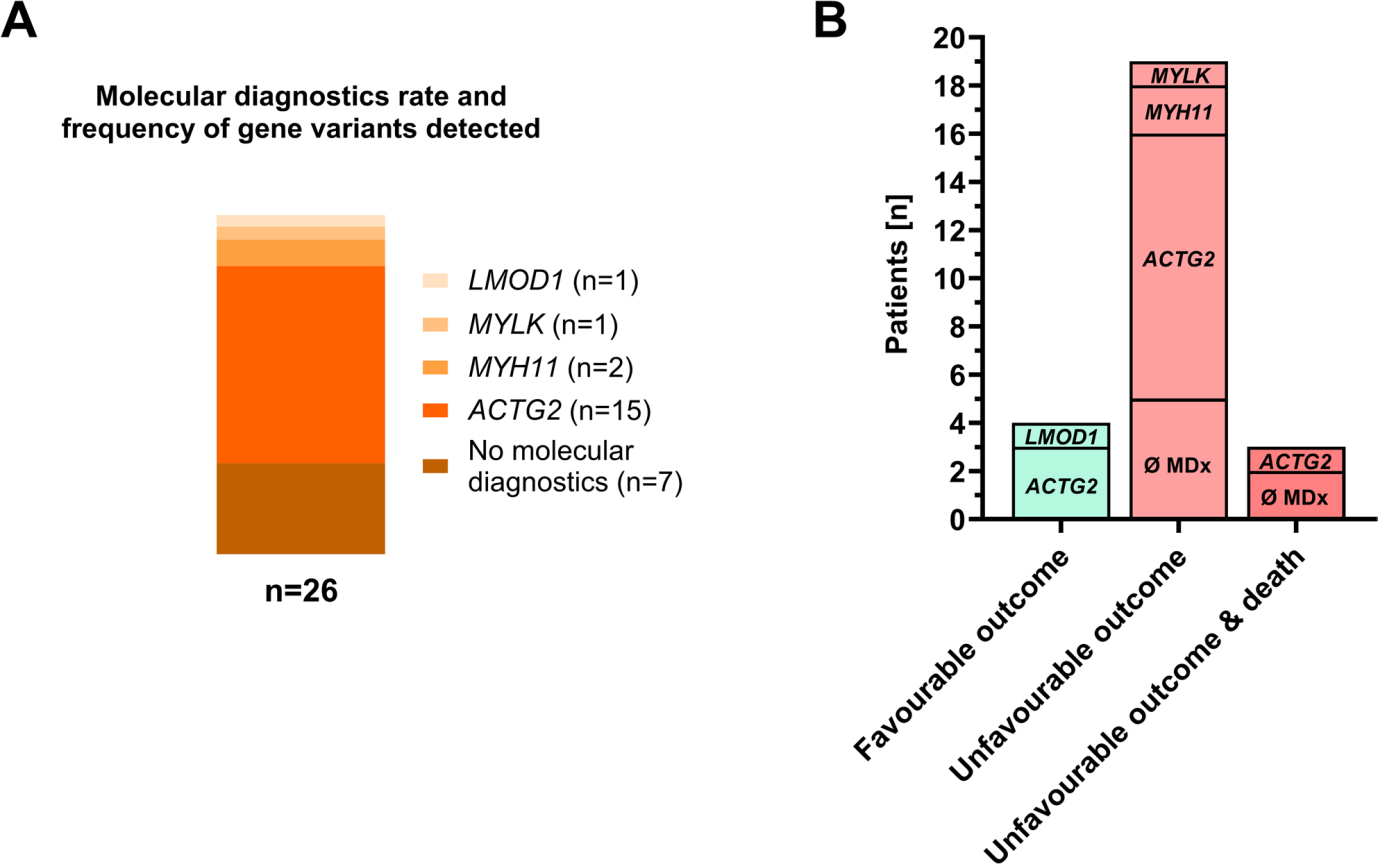
Molecular diagnostics rate, results and respective patient outcome in the studied MMIHS cohort. (**A**) Frequency of molecular diagnostics and affected gene distribution in the cohort of MMIHS. Gene variants with frequencies are indicated, while in 7 patients no molecular diagnostics was performed. (**B**) Distribution of affected genes per patient according to clinical outcome in MMIHS. Outcomes were classified as favorable outcome (complete bowel continuity + enteral nutrition), unfavorable outcome (ostomy + parenteral nutrition), and unfavorable outcome & death. The number of patients per affected gene and those without molecular diagnostics (Ø MDx) is shown (*n* = 26)

Molecular diagnosis was performed in 19 of 26 cases, and all 19 cases identified a gene variant associated with visceral myopathy. *ACTG2* was the most prevalent gene variant identified (15/19), followed by *MYH11* (2/19), *MYLK* (1/19), and *LMOD1* (1/19) (Fig. 3A; Table 1). A missense mutation was identified in 14 of 15 patients with *ACTG2* variants. For one patient, the detailed mutation could not be identified retrospectively. Among *ACTG2* missense mutations, R257C was the most common (*n* = 4), followed by R257H and R178C (both *n* = 3), R40H (*n* = 2), and R63Q and P39R (both *n* = 1).

The patients were grouped into favorable and unfavorable outcomes defined by the presence of an ostomy or parenteral nutrition dependency (unfavorable outcome). Four patients exhibited favorable outcomes (3 x *ACTG2* variant, 1 x *LMOD1* variant), and 22 had unfavorable outcomes (7 x no molecular diagnostics, 12 x *ACTG2* variant, 2 x *MYH11*, 1 x *MYLK* variant) (Fig. 3B; Table 1). Three patients with an unfavorable outcome died (Fig. 3B; Table 1), one due to complications of intestinal transplantation, one due to liver failure in IFALD, and one due to a combination of IFALD and multiple infections.

### Genotype-phenotype correlation in *ACTG2* variants

**Fig. 4:**
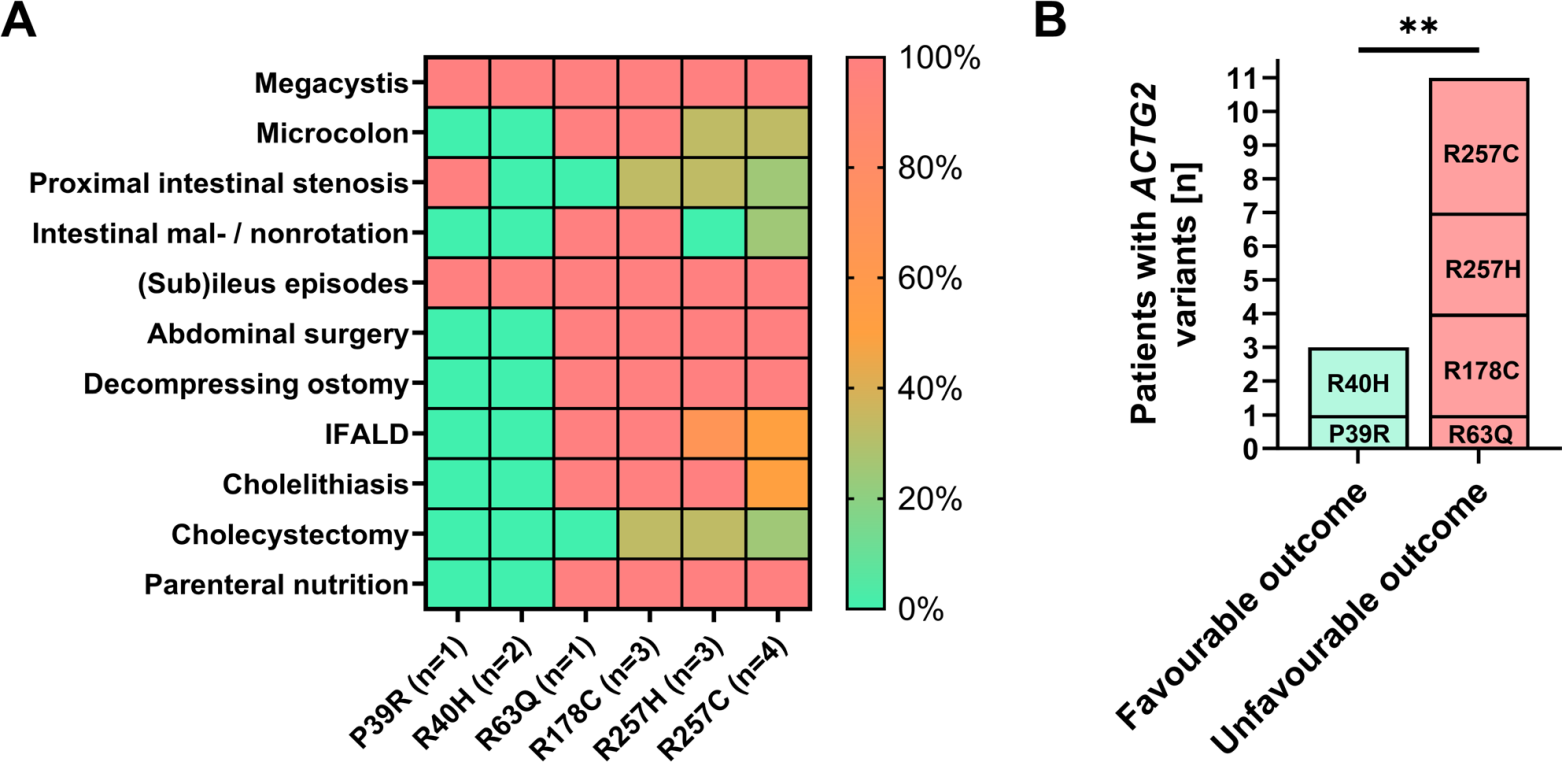
Genotype-phenotype correlation of *ACTG2* variants in the studied MMIHS cohort. (**A**) Frequency of disease-specific clinical features and required treatment measures in *ACTG2* variants. The abundancy of respective feature found in patients with respective *ACTG2* variants (*n* = 14) are indicated as heat map as percentages. Group comparisons between favorable outcome variants P36R and R40H versus unfavorable outcome variants R63Q, R178C, R257H and R257C are found in Table 2. (**B**) Missense substitutions in *ACTG2* are shown comparing unfavorable (ostomy + parenteral nutrition) versus favorable outcome (complete bowel continuity + enteral nutrition) in MMIHS. Number of patients are indicated. Group differences were analyzed by Exact Fisher´s test (**, *p* ≤ 0.01)

*ACTG2* variants were the predominant genetic cause of MMIHS in our cohort, but individual clinical symptoms and disease severity varied significantly. To assess the severity of specific missense mutations in *ACTG2*, we correlated the 14 patients with determined *ACTG2* genotypes of our cohort with their respective phenotypes. While megacystis and (sub)ileus episodes, as an expression of intestinal hypoperistalsis, were present in all *ACTG2* variants and individuals, other features significantly varied between specific variants (Fig. 4A; Table 2). Patients with the *ACTG2* variant R40H or P39R did not exhibit other disease-specific features (except of a proximal intestinal stenosis for the P39R patient), did not require abdominal surgery or parenteral nutrition, and were therefore the only *ACTG2* variants grouped for favorable outcome (Fig. 4A and B). The other *ACTG2* variants (R63Q, R178C, R257H, and R257C) found in our cohort were significantly more likely to be associated with the need for decompression stomata (100%), parenteral nutrition (100%), and hepatobiliary complications (IFALD, 72.2%; cholelithiasis, 81.8%) than R40H and/or P39R (Fig. 4A; Table 2). Thus, *ACTG2* variants R63Q, R178C, R257H, and R257C resulted in significantly more unfavorable outcomes for MMIHS than R40H and P39R (Fig. 4B; Table 2). When comparing R257H and R257C with R63Q and R178C, there seems to be a trend for the latter two to possess most MMIHS-specific features, since microcolon, mal-/ non-rotation, and hepatobiliary complications were found most consistently in these variants (Fig. 4B; Table 2). Therefore, genotype-to-phenotype severity and likelihood for an unfavorable outcome in MMIHS by *ACTG2* variants can be ranked as R178C > R63Q > R257H > R257C > P39R > R40H in this cohort.

**Table 2:** Genotype-phenotype correlations of *ACTG2* variants

Phenotypic feature \ genotypic variant	ACTG2–All (All variants)	ACTG2–1		ACTG2–2				*p*-value ACTG2–1 vs. ACTG2–2
		P39R	R40H	R63Q	R178C	R257H	R257C	
Patients [n, (%)]	14 (100%)	1 (7.1%)	2 (14.3%)	1 (7.1%)	3 (21.4%)	3 (21.4%)	4 (28.6%)	***p*** **= 0.007**
Female [n, (%)]	8/14 (57.1%)	1/1 (100%)	2/2 (100%)	1/1 (100%)	2/3 (66.7%)	0/3 (0%)	2/4 (50%)	*p* = 0.209
Age [months, median (range)]	96 (6-176)	161 (N/A)	79 (13–158)	6 (N/A)				*p* = 0.555
Favorable outcome	3/14 (21.4%)	1/1 (100%)	2/2 (100%)	0/1 (0%)	0/3 (0%)	0/3 (0%)	0/4 (0%)	***p*** **= 0.003**
Unfavorable outcome	11/14 (78.6%)	0/1 (0%)	0/2 (0%)	1/1 (100%)	3/3 (100%)	3/3 (100%)	4/4 (100%)	***p*** **= 0.003**
Megacystis	14/14 (100%)	1/1 (100%)	2/2 (100%)	1/1 (100%)	3/3 (100%)	3/3 (100%)	4/4 (100%)	N/A
Microcolon	5/12 (41.7%); missing data = 2	0/1 (0%)	0/2 (0%)	1/1 (100%)	2/2 (100%); missing data = 1	1/3 (33.3%)	1/3 (33.3%); missing data = 1	*p* = 0.205
Decompressing stoma	11/14 (78.6%)	0/1 (0%)	0/2 (0%)	1/1 (100%)	3/3 (100%)	3/3 (100%)	4/4 (100%)	***p*** **= 0.003**
Proximal intestinal stenosis	4/14 (28.6%)	1/1 (100%)	0/2 (0%)	0/1 (0%)	1/3 (33.3%)	1/3 (33.3%)	1/4 (25%)	*p* = 1.000
Cholelithiasis	8/14 (57.1%)	0/1 (0%)	0/2 (0%)	1/1 (100%)	3/3 (100%)	3/3 (100%)	2/4 (50%)	***p*** **= 0.027**
Parenteral Nutrition	11/14 (78.6%)	0/1 (0%)	0/2 (0%)	1/1 (100%)	3/3 (100%)	3/3 (100%)	4/4 (100%)	***p*** **= 0.003**
IFALD	8/14 (57.1%)	0/1 (0%)	0/2 (0%)	1/1 (100%)	3/3 (100%)	2/3 (66.6%)	2/4 (50%)	*p* = 0.055

IFALD, intestinal failure-associated liver disease. Exact Fisher´s test or Mann-Whitney-U-Test for group comparison. N/A, not applicable. Respective *p* values are indicated; A *p* < 0.05 was considered as significant difference and is displayed in bold

One male patient in this cohort had a homozygous *LMOD1* missense mutation with an overall favorable outcome at the last follow-up and featured megacystis, partial duodenal stenosis, status after temporary PN support currently under enteral nutrition, and cholelithiasis (Table 1). Both patients with *MYH11* variants were female and showed an unfavorable outcome with PN dependency and decompression ostomy due to recurrent ileus episodes (Table 1). The only patient with a *MYLK* variant in our cohort presented with megacystis, microcolon, duodenal stenosis and persistent ileus. She was managed by proximal jejunostomy and parenteral nutrition and developed IFALD and cholelithiasis (Table 1).

## Discussion

MMIHS is an exceedingly rare and severe congenital disorder. We present the first report of pediatric-onset MMIHS from the largest national referral center for pediatric intestinal failure in Germany. We analyzed the phenotypic characteristics, genotype-phenotype correlations, and medical and surgical management in this second largest case series of patients with MMIHS worldwide [13]. As one patient (#25) was published previously as a short report [12], this study adds 25 new patients to the literature.

Our study highlights a high rate of proximal intestinal stenosis, disorders of intestinal rotation and liver involvement (IFALD and cholelithiasis) in MMIHS. While megacystis and hypoperistalis are common, microcolon is less common in our cohort. Prenatal ultrasound effectively provides a suspected diagnosis for MMIHS which should lead to genetic diagnostics. *ACTG2* variants dominate in MMIHS but show significant variations of disease severity requiring genotype-based medical advice. Current treatment measures including high ostomy and PN enabled long-term survival of 88% in our cohort.

From the eponymous symptoms of MMIHS, we found that megacystis and defective intestinal peristalsis were present in 96% and 100% of our cohort, respectively. In contrast, a microcolon was observed less frequently (57%) and has been associated with unfavorable outcome in recent analyses [13, 19]. Thus, the absence of a microcolon does not exclude the diagnosis of MMIHS. In our cohort, a high rate of proximal bowel stenosis was observed (35%; 6 duodenal and 3 jejunal), with diagnosis made upon endoscopy or imaging. A mechanical stenosis is not described as a characteristic feature of MMIHS. However, segmental stenosis has been reported [8]. Only six patients in our cohort with presumed stenosis required resection. Bowel segments may appear stenotic due to absent peristalsis rather than true mechanical obstruction. As a result, and even though nonobstructive gastrointestinal dysmotility is a classic feature of MMIHS, we recommend active screening for intestinal stenosis in patients with MMIHS. Since intestinal malrotation or non-rotation was present in 39%, it can be a contributor of impeded intestinal transit in MMIHS which should be actively screened and surgically treated if appropriate. MMIHS is suspected if megacystis is detected during prenatal ultrasound [4, 20]. In our cohort, a fetal megacystis with or without hydronephrosis was noted in 96% of cases, while some kinds of sonographic abnormalities were found in all patients later diagnosed with MMIHS. Since LUTO is an important differential, patients with prenatal signs of visceral myopathy but no LUTO should undergo genetic diagnostics. Reduced enteral intake and parenteral nutrition are risk factors for cholelithiasis. Nevertheless, the release of bile into the intestinal tract relies on smooth muscle contractions of the gallbladder, which, as a hollow organ, does express *ACTG2* [21]. Since the reported frequency of cholelithiasis in children with intestinal failure (mainly short bowel syndrome) managed with long-term PN is 21% [22], the effects of visceral myopathy on gallbladder function may explain the high rate of cholelithiasis (65%) observed in our MMIHS cohort. We propose that in MMIHS, visceral myopathy impairs gallbladder contractility, causing bile stasis and further increasing the risk of gallstones, in addition to the usual risks associated with intestinal failure, parenteral nutrition, and reduced enteral stimulation.

Five patients (19%) in our cohort required cholecystectomy due to symptomatic cholelithiasis. Of note, in patients with *ACTG2* variants other than P39 or R40 mutations cholecystolithiasis was significantly more likely suggesting a cumulative effect of PN and visceral myopathy in MMIHS cholelithiasis. Therefore, contrary to the recommendations for intestinal failure by short bowel syndrome [23, 24], we suggest proactively considering elective cholecystectomy in patients with MMIHS during (elective) abdominal surgery to prevent complications such as cholecystitis.

A female-to-male ratio of MMIHS cases as high as 2.5:1 has been described, although some authors dispute this finding and suggest a reporting bias [8, 19, 25]. Our study revealed a modest female predominance (62%), which aligns with the findings of the aforementioned systematic review that encompassed 103 patients (52.4% female) [19]. While female sex has been linked to poorer outcomes [19], our data suggest this association is inconclusive. Thus, the role of female gender as a negative outcome predictor in MMIHS is questionable. The patients with favorable outcome were the only three individuals with P39 or R40 *ACTG2* variants. All other *ACTG2* variants were associated with unfavorable outcome (severity: R178 < R63 < R257), requiring high intestinal decompression stoma and parenteral nutrition. A similar severity spectrum was reported for arginine missense substitutions with R178 >R63 >R257 >R40 in the largest cohort of 28 patients with *ACTG2*-dervied MMIHS [13]. However, in their cohort (which included patients from 1999 to 2001 and 2013–2019) poor outcomes were defined as total parenteral nutrition dependence, death, or transplantation with a rate of 57.1% for *ACTG2* variants. In our cohort, 19 from 26 patients underwent genetic diagnostics, all of which were positive for MMIHS-causing gene variants, predominantly affecting *ACTG2*. Yet, other gene variants occur and can present different clinical features compared to *ACTG2*-mediated MMIHS. We found two patients possessing a *MYH11* and one patient with a *MYLK* and a LMOD1 variant, respectively, differing in both clinical features (e.g., no megacystis in one *MYH11* patient) and subsequent management (e.g., no ostomy in the *LMOD1* patient) from *ACTG2* variants. While case reports on such rare variants are especially lacking, we show that the overall management strategies for MMIHS with a generous indication for a high decompressing ostomy and subsequent PN is successful in these variants, as well. However, management remains an individual decision according to the clinical features. We show that phenotype strongly depends on genotype with crucial clinical differences between both MMIHS-causing variants and within *ACTG2* variants. Thus, genetic diagnostics is cornerstone for both successful diagnosis and management and should be advised in all patients with suspected MMIHS.

Since megacystis detected on prenatal ultrasound provides a reliable early indicator of MMIHS, early genetic diagnosis is feasible and could help prevent complications such as ileus episodes. Moreover, knowledge of the underlying genotype – particularly *ACTG2* variants at P39 and R40 – may guide prenatal counseling by offering realistic prognostic expectations and enable individualized management strategies, including surgical approaches.

Historically, MMIHS was regarded as a fatal condition, with the majority of patients dying within the first year of life [1, 26]. Major causes of death in MMIHS are reported to be (uro)sepsis and liver failure, both associated with PN-related complications [8]. As survival rates in MMIHS have improved with recent advances in intestinal rehabilitation, genotype-to-phenotype analyses correlating death or transplantation as outcome parameters may be biased, particularly in historical analyses. Therefore, we defined the presence of an intestinal decompression stoma and the need for PN as a more disease-specific outcome that reflects severity. With this treatment approach, the overall long-term prognosis in our cohort was good, with long-term survival in 23 patients (88%). However, a potential referral bias must be considered, as more complex cases are likely to be referred to our reference center. Conversely, a higher index of suspicion may also have facilitated recognition and diagnosis of less severe cases. Regardless, the survival rate in our cohort contrasts with the historic 19.7% of Gosemann & Puri, who systematically reviewed 227 MMIHS cases published between 1976 and 2011 [8]. This striking discrepancy compared with our cohort, which spans the period from 2010 to 2025, likely reflects overall improvements in the care of patients with chronic intestinal failure, including advances in parenteral nutrition and surgical approaches, as well as better prevention of complications such as central line-associated bloodstream infections, venous thrombosis, and intestinal failure-associated liver disease [27]. This observation underscores the importance of, and supports the recommendation for, managing children with intestinal failure in experienced centers with intestinal rehabilitation programs [28].

Our study was limited by its retrospective design and small sample size due to the rarity of the disease, which restricts statistical power for detailed analysis. Another limitation is that molecular confirmation was not available for all patients. However, genotype-phenotype analyses included only those with genetic testing. Our study encompasses a time span of > 10 years which included changes in guidelines and e.g. PN recommendations for intestinal failure.

## Conclusions

This study is the second largest case series of patients with MMIHS and provides additional evidence regarding the phenotypic and genotypic characteristics of patients with pediatric-onset MMIHS. While megacystis and defective intestinal peristalsis were present in most patients, a microcolon was less common. A clinically relevant rate of small bowel stenosis, disorders of intestinal rotation and cholelithiasis was observed. *ACTG2* was the most prevalent target of gene variation, and patients with *ACTG2* mutations at P39 and R40 had significantly more favorable outcomes than those with other variants. Current and profound management of PN, catheter handling to prevent infections, and surgical placement of high decompressing ostomy adapted to the individual’s genotype can result in beneficial long-term survival in MMIHS (e.g., 88% in our cohort). Therefore, determining the genotype at diagnosis is essential for management and counselling. Prospective multicenter registries are needed to confirm our data and provide more insights into the genotype-to-phenotype characteristics of individuals with MMIHS.

## Data Availability

The datasets generated and/or analyzed during the current study are not publicly available due to patient confidentiality but are available from the corresponding author on reasonable request.

## Abbreviations

IFALD: Intestinal Failure-Associated Liver Disease
MMIHS: Megacystis-Microcolon-Intestinal Hypoperistalsis Syndrome
PN: Parenteral Nutrition
PNDI: Parenteral Nutrition Dependency Index
